# Neurocognitive Impairment and Social Cognition in Parkinson’s Disease Patients

**DOI:** 10.3390/neurolint16020032

**Published:** 2024-04-16

**Authors:** Triantafyllos Doskas, Konstantinos Vadikolias, Konstantinos Ntoskas, George D. Vavougios, Dimitrios Tsiptsios, Polyxeni Stamati, Ioannis Liampas, Vasileios Siokas, Lambros Messinis, Grigorios Nasios, Efthimios Dardiotis

**Affiliations:** 1 Department of Neurology, Athens Naval Hospital, 11521 Athens, Greece; dantevavougios@hotmail.com; 2 Department of Neurology, General University Hospital of Alexandroupoli, 68100 Alexandroupoli, Greece; vadikosm@yahoo.com (K.V.); tsiptsios.dimitrios@yahoo.gr (D.T.); 3 251 Airforce General Hospital, 11525 Athens, Greece; kntoskas@yahoo.gr; 4 Department of Neurology, Faculty of Medicine, University of Cyprus, 1678 Lefkosia, Cyprus; 5 Department of Respiratory Medicine, Faculty of Medicine, School of Health Sciences, University of Thessaly, 41500 Larissa, Greece; 6 Department of Neurology, Faculty of Medicine, School of Health Sciences, University of Thessaly, 41110 Larissa, Greece; tzeni_0@yahoo.gr (P.S.); liampasioannes@gmail.com (I.L.); bill_s1983@hotmail.com (V.S.); ebsdar@gmail.com (E.D.); 7 School of Psychology, Laboratory of Neuropsychology and Behavioural Neuroscience, Aristotle University of Thessaloniki, 54124 Thessaloniki, Greece; lmessinis@psy.auth.gr; 8 Department of Speech and Language Therapy, School of Health Sciences, University of Ioannina, 45500 Ioannina, Greece; nasios@uoi.gr

**Keywords:** Parkinson’s disease, cognitive impairment, cognitive function, social cognition

## Abstract

In addition to motor symptoms, neurocognitive impairment (NCI) affects patients with prodromal Parkinson’s disease (PD). NCI in PD ranges from subjective cognitive complaints to dementia. The purpose of this review is to present the available evidence of NCI in PD and highlight the heterogeneity of NCI phenotypes as well as the range of factors that contribute to NCI onset and progression. A review of publications related to NCI in PD up to March 2023 was performed using PubMed/Medline. There is an interconnection between the neurocognitive and motor symptoms of the disease, suggesting a common underlying pathophysiology as well as an interconnection between NCI and non-motor symptoms, such as mood disorders, which may contribute to confounding NCI. Motor and non-motor symptom evaluation could be used prognostically for NCI onset and progression in combination with imaging, laboratory, and genetic data. Additionally, the implications of NCI on the social cognition of afflicted patients warrant its prompt management. The etiology of NCI onset and its progression in PD is multifactorial and its effects are equally grave as the motor effects. This review highlights the importance of the prompt identification of subjective cognitive complaints in PD patients and NCI management.

## 1. Introduction

Parkinson’s disease (PD) is a chronic heterogeneous neurodegenerative disease of unknown etiology that affects the central nervous system (CNS) [1]. It is the second most reported neurodegenerative disease after Alzheimer’s disease (AD). PD is characterized by the degeneration of dopaminergic neurons in the substantia nigra and a subsequent decrease in dopamine production [2] and is attributed to a complex interplay between immunological, aging, genetic, and environmental factors [3,4,5,6,7,8]. Idiopathic PD, which is the most frequently reported PD subtype, is an age-associated disease that affects 1% of patients over 65 years of age [9]. Younger people, below the age of 55 years, can also be affected (early-onset PD) [3]. 

The clinical symptoms of PD are heterogeneous, depending on onset age, comorbidities, and treatment (both dopaminergic treatment and treatment for comorbidities) [10,11,12]. PD clinically presents with motor symptoms, including bradykinesia, rigidity, and resting tremor, and, at a more advanced stage, postural instability. Based on motor symptoms, PD patients are broadly distinguished into those with the tremor-dominant (TD) subtype, with postural instability and gait disorder (PIGD or the akinetic-rigid-AD subtype), or their combination (indeterminate subtype) [10,12,13]. PD subtypes differ in biochemical and imaging features, as well as in disease progression [12,13,14,15]. 

Non-motor symptoms are prevalent in the prodromal phase of PD and may precede motor symptoms by up to 20 years [16,17,18]. These include depression, gastrointestinal problems (such as constipation), anxiety disorders, olfactory dysfunction, sleep disturbances, nausea, neurocognitive impairment (NCI), social brain dysfunction, fatigue, apathy, and erectile dysfunction [16,19]. Some of the symptoms have a dopaminergic etiology, namely sleep disturbance, erectile dysfunction, and depression [20,21]. Neural cognition encompasses a wide range of functions that include episodic memory, working memory, attention, language, the speed and efficiency of information processing, attention, orientation, executive function (EF), and verbal fluency. Neurodegenerative diseases are characterized by a long prodromal period of non-motor symptoms, including NCI due to neurodegeneration in the parts of the brain that control the corresponding neurocognitive functions, which precede motor symptoms [22]. However, the severity and type of neurocognitive deficits differ among neurodegenerative diseases and can be exploited for differential diagnostic purposes [23,24]. Deficits in attention, concentration, thinking, planning, language, verbal fluency, and memory are frequently reported in PD patients [22,25], whereas attention and working memory are the most frequently reported neurocognitive deficits in newly diagnosed PD patients [26,27]. Deficits in episodic memory, EF, language, visuospatial abilities, and attention/executive memory were detected in 34% of newly diagnosed PD patients without suspected NCI, depression, or neuropsychiatric problems [28]. PD-associated NCI is the result of the interaction of several underlying factors [29], namely aging, which is the most important risk factor for the occurrence of dementia in the general population, and PD-related NCI [30,31]. Late PD onset age correlates with a higher risk of dementia [23,24]. Additionally, considering dopamine’s contribution to social and neural cognition, its reduced production and transmission make PD patients vulnerable to social cognition impairment and neurocognitive deficits, respectively [32,33,34]. The neurocognitive decline seen in PD is heterogeneous both in terms of symptoms and its progression. It may appear before motor symptom onset and coexist with hallucinations as well as sleep and autonomic nervous system disorders [22,35,36]. This heterogeneity is also attributed to genetic variations that have enabled genotype–phenotype associations to be made [37,38]. PD NCI ranges from subjective, mild cognitive impairment (MCI-PD) to PD dementia (PDD) with concordant severe effects on the daily life of PD patients [22]. NCI deficits are also evident in PD patients with normal cognition (PD CN). NCI onset may affect the severity of other non-motor symptoms, such as depression [39]. 

Considering that PD mainly affects the elderly, NCI was underestimated in the past, because it was attributed mainly to aging and not the disease itself and thus escaped patients’ attention. The availability of validated assessment tools, both for global cognition and individual neurocognitive functions, and the establishment of criteria for the diagnosis of MCI-PD and PDD have contributed to the investigation of the prevalence of NCI in PD patients and its impact [40,41,42]. In addition to dedicated tests, neuropsychological batteries are commonly employed to determine the functioning of many individual neurocognitive domains [42]. 

Common to all neurodegenerative diseases, non-motor symptoms confer a significant burden on PD patients and their carers and contribute to disability and overall impaired quality of life (QoL), in addition to increased healthcare costs [20,24,40,43,44]. PD-related morbidity and mortality are increasing faster compared to other neurodegenerative diseases [3]. The awareness and prompt detection of prodromal non-motor symptoms, namely NCI, may provide a window for effective interventions, such as cognitive rehabilitation, for the effective management of PD before motor symptom onset. This is particularly important because there is no confirmed neuroprotective treatment for PD that slows, inhibits, or reverses its progression. PD treatment involves levodopa, dopaminergic therapy, or deep brain stimulation and is mainly aimed at improving motor symptoms [45]. There is also no cure for PDD, and memory problems can be managed with cholinesterase inhibitors. Non-pharmacological approaches, such as exercise and physical therapy, can be beneficial at all stages of the disease. The introduction of assessment criteria for determining NCI and the development of a range of valid tools to evaluate the neurocognitive status of PD patients [46,47] have been valuable for delaying, preventing, or predicting the progression of PD to PDD. The importance of early diagnosis is evident from the growing number of studies suggesting that NCI can not only be used prognostically to determine the severity of the motor symptoms of PD, especially freezing, but also potential long-term treatment decisions, such as an increase in levodopa dose [48]. The latter parameter correlates with visuospatial cognitive functions and verbal memory [48]. 

The purpose of this review is to investigate NCI prevalence and phenotypes in PD as well as the effect of non-motor and motor symptoms on the prognosis and progression of PD NCI. The heterogeneity of PD symptoms poses a challenge. As NCI precedes motor symptoms, preventing or delaying the progression of NCI may confer a protective effect on the progression of the motor manifestations of PD. Due to the interconnection of motor and neurocognitive symptoms, exploiting non-motor symptoms diagnostically may allow for the prompt identification of PD patients who are at greater risk of PDD. Delaying or preventing PDD and its subsequent effects on the daily functioning of PD patients and their carers is of paramount importance.

## 2. Materials and Methods

A thorough search of the literature on PubMed/Medline for articles published up to March 2023 using the following keywords: Parkinson, Parkinson’s, Parkinson’s disease, cognitive impairment, cognition, cognitive decline, mild cognitive impairment, subjective cognitive complaints, social cognition, alexithymia, and theory of mind was conducted. The retrieved publications were then searched for further pertinent references, if applicable. The sources that fulfilled the inclusion criteria were case series and cohort studies as well as systemic reviews, and meta-analyses. The results of systematic reviews and meta-analyses contributed greater validity compared to individual studies. Small sample size studies or case series studies were excluded due to the inherent limitations of a limited population sample and increased heterogeneity among patients as well as the lack of uniformity in assessments used. Studies reporting correlations between imaging and NCI were out of scope and were also excluded. The publications that were used were available in English and fulfilled PRISMA guidelines. 

There are limited longitudinal studies on the progression of NCI over time. Most of the available data are mainly from cross-sectional studies. Longitudinal studies that include successive assessments of the same patients over time are considered more reliable than cross-sectional studies. Another limitation was the lack of consistency in the methodology and/or tools that were used to determine subjective neurocognitive decline or the neurocognitive functions that were affected. A single question on the presence or absence of a subjective decline in memory, specifically or subjective, and neurocognitive complaints (SCCs) in general was used in some studies, whereas validated tools were used in others [49]. Despite the limitation of a small sample size, studies that included PD patients with various states of neurocognitive functioning ranging from PD-NCI to MCI and PDD have enabled comparisons among them and contributed evidence on qualitative differences. Although SCCs may less accurately reflect NCI, they may be valuable prognostically.

## 3. Risk Factors for Neurocognitive Impairment in PD Patients

NCI can be diagnosed in the prodromal period of PD, at PD onset, or later in the course of the disease. Aging and aging-related pathologies may differentially affect PD motor and non-motor manifestations [50]. Aging-related pathologies, such as anemia and visual impairment, have also been associated with an increased risk of NCI and dementia in the general population [51,52]. Despite symptom heterogeneity, PD onset at an older age was positively related to the degree of NCI [12,13,14,15,53,54,55,56]. In the longitudinal prospective study by Pigott et al. [57], NCI in patients with baseline CN, with a mean age of 68.8 years and predominantly male (63%), who had PD for an average of 5 years, MCI, or PDD was observed in 81% of patients at 6 years. Overall, NCI was observed in 8.5% of patients in the first year, increasing to 47.4% in the sixth year. The percentage of MCI-PD patients was 7.8% in the first year, 18.5% at 2 years, 28% at 3 years, 36.1% at 4 years, and 43% at 6 years. Accordingly, the percentage of patients who developed PDD was 0.7% in the first year, 3.5% at 2 years, 7.5% at 3 years, 12.9% at 4 years, and 28% at 6 years. Male gender and severity of motor symptoms were statistically significantly associated with future NCI onset [57]. Vascular comorbidities, which commonly affect patients of age > 60 years old, such as dyslipidemia, in addition to autonomic nervous system dysfunction and the effects of the administered dopaminergic therapy, may introduce variability in blood pressure, which in turn could contribute to the development of white matter hyperintensities and NCI due to arterial stiffness, microvascular atherosclerosis, and vascular endothelial dysfunction [58]. It has therefore been proposed that orthostatic hypotension, which is commonly observed in PD patients, may be associated with NCI [59]. Additionally, prolonged dopaminergic treatment, male sex, affective disorders (e.g., depression), and potential treatment for depression (benzodiazepines, anticholinergic agents, and tricyclic antidepressants), as well as genetic and environmental parameters, can contribute to PD NCI. Systemic inflammatory responses due to oxidative stress also potentially contribute to the development of white matter hyperintensities and may be associated with attention, executive, and memory deficits [60,61], though the incidence or severity of white matter hyperintensities was comparable in PD patients and age-matched healthy subjects, suggesting that neurocognitive deficits may precede the appearance of PD-induced imaging changes [62]. However, the severity of white matter hyperintensities in anemic PD subjects was more unfavorable compared to healthy subjects or PD patients with intellectual impairment [62]. 

The effect of non-motor symptoms on NCI onset or deterioration warrants investigation. Furthermore, anxiety disorders confer deficits in verbal memory and EF in PD patients [63,64], whereas sleep disorders contribute longitudinally to NCI in PD [65]. The association between apathy, affective disorders, and NCI is being increasingly investigated [49,66,67,68,69,70]. Apathy affects approximately 40% of PD patients and may occur independently of depression or NCI, although pure apathy is rarely reported and co-presents with depression and/or anxiety [21,66,67,68,70,71]. A systematic review and meta-analysis reported that depression in PD patients was associated with disease duration, PIGD rather than the TD kinetic subtype, anxiety, apathy, fatigue, daytime somnolence, NCI, and GBA1 mutation [21]. NCI in early-onset PD patients and disease duration correlate significantly with depression onset in afflicted patients [39]. A cross-sectional study of older PD adults versus healthy controls reported that apathy and not depression was the only predictor of EF, anxiety was the only predictor of attention/working memory, or both depression and apathy correlated with immediate memory deficits, which remained even after adjustment for depression [69,72]. It was thus proposed that apathy may precede NCI in PD, occurs secondarily to mood disorders, and contributes to NCI by interfering not only with EF but with other neurocognitive functions as well [21,66,67,68,69,70,71,72]. In addition to memory deficits, apathetic PD patients with mild ED and no global NCI may have information processing speed (IPS) deficits [73]. In the systematic review by den Brok et al. (2020), the prevalence of apathy in PD patients without depression or NCI was 22.6%, and the prevalence of depression was 57.7% in apathetic PD patients without NCI compared to 21.9% in non-apathetic patients [66]. Compared to non-apathetic, non-demented, and non-depressed patients, apathetic PD patients had higher global NCI and ED and, for 18 months, quicker progression to PDD in the small longitudinal study by Dujardin et al. (2009) [67]. NCI, global and presented with deficits in EF and memory, was significantly greater in non-demented apathetic subjects than in non-apathetic subjects [67]. 

Another factor that may further complicate neurocognitive assessments in patients with neurodegenerative diseases is anosognosia. Anosognosia is defined as a progressive decrease in self-awareness or impaired insight for cognitive or behavioral deficits [74]. It therefore contributes to the underestimation of NCI in PD or AD patients, particularly in patients with objective neurocognitive decline (MCI and dementia) [27,74,75,76]. Anosognosia has major clinical implications for self-management, compliance with treatment, and caregiver burden for both AD and PD patients [74,75,77,78,79]. Anosognosia is inversely correlated with depression in AD and, in the majority of published studies, PD [27,75,76,78,79]. Yoo et al. (2020) reported that cognitive anosognosia (CA) was detected in approximately 22% of drug-naïve PD patients. The PD-CA subgroup (objective neurocognitive decline but no SCCs) in the study had greater NCI in frontal EF, attention, working memory, IPS, and cognitive flexibility and lower depression compared to PD subgroups with normal neural cognition (with neither SCCs nor objective neurocognitive decline) or with cognitive underestimation (with SCCs but no objective neurocognitive decline) [27]. Thus, SCCs could mediate the association between cognitive anosognosia and depression in PD patients [27]. 

Large-scale proteomic analysis of the human brain has identified proteins that contribute to stable cognitive trajectory in advancing age, regardless of neuropathology [80]. Genome-wide association studies have provided firm evidence of genotype–phenotype associations in PD patients with NCI [38,81,82]. Other than the contribution of potential vascular comorbidities, polymorphisms in genes associated with vascular and metabolic factors, including catechol-O-methyltransferase, (COMT), apolipoprotein E (APOE), and vascular endothelial growth factors (VEGF), have an established effect on neurocognitive deficits [31,38,58,83]. A recent case–control genome-wide association study on PD patients with a mean follow-up of 4.2 years demonstrated that the APOE E4 allele contributes to progressive NCI in PD [37]. The association between the APOE E4 mutation and faster mental decline and more unfavorable Hoehn and Yahr staging has also been confirmed, paving the way for its use as a predictor of NCI in PD [84]. Meta-analyses have also confirm that this gene is a risk factor for the development of PDD, regardless of race, allowing for its potential universal use in the context of PD prognosis [85]. Furthermore, SNCA (α-synuclein) mutations and polymorphisms of the SNCA promoter are consistent with neurocognitive deficits/decline, though not systematically with adverse motor outcomes, regardless of onset age [4,38,86,87,88,89,90,91,92,93,94,95,96,97]. The SNCA polymorphism rs894280 correlates with attention and visuospatial deficits in idiopathic PD patients [89]. SNCA (alpha-synuclein) triplications are consistent with early-onset PD (<55 years) and rapid symptom progression that includes a high rate of psychotic symptoms and depression, early onset of NCI, and autonomic dysfunction [38,81]. Mutations that are associated with decreased NCI burden and behavioral symptoms in PD patients have also been identified, such as LRRK2 mutations [98].

Apart from the contribution of risk factors, the protective effect of neurocognitive reserve has been investigated in various neurodegenerative diseases [99,100]. A meta-analysis of longitudinal studies on the contribution of higher educational levels in cognitive reserve in PD patients demonstrated a correlation with overall better general cognitive function, EF, memory, and IPS [100]. There was no significant association between educational levels and visuospatial function or language in PD patients, whereas a negative association between educational level and the risk of longitudinal progression to MCI-PD was identified [100].

## 4. PD Neurocognitive Phenotypes

### 4.1. Subjective Neurocognitive Decline

Subjective neurocognitive decline (SCD) refers to early changes in neurocognitive functioning that are most commonly self-reported by PD patients or determined by clinicians and are not necessarily detected with neuropsychological assessments. SCD is increasingly investigated in older adults, regardless of underlying neurodegenerative diseases, as well as in patients with neurodegenerative diseases. Longitudinal studies of people with SCCs that are not associated with neurodegenerative diseases confirm that SCCs are associated with an increased risk of future dementia or MCI [101], whereas others confirm that SCCs in memory reflects depressive symptoms and is not associated with objective NCI [102]. SCD is also common in AD patients and newly diagnosed PD patients [103], with statistically significant associations with an increased risk of MCI and dementia [49,101]. Increasing evidence over the last decade has confirmed that SCCs are commonly reported by PD patients, regardless of neurocognitive status (PD-CN, MCI-PD, and PDD), diagnosis, or disease onset (early- and late-onset PD patients) [26,43,49,104,105,106,107]. The prevalence of SCCs in MCI-PD and PD-CN patients ranges from 10.7–84% and 6.3–83%, respectively [26,104,107,108]. This broad range reflects both the heterogeneity of the patients among studies and the use of different assessment tools for determining SCD. SCCs concern deficits not only in memory but also in other neurocognitive functions, including attention, working memory, visual memory, visuospatial function, EF, IPS, and semantic or phonemic fluency [26,43,49,104,106,109,110]. The severity of SCCs increases with increasing NCI, with PDD patients being more severely affected than MCI-PD or PD-CN [26,105,106]. It has thus been proposed that the severity of SCCs may be used to discriminate PDD versus non-demented PD patients [105]. Potential imaging, molecular, or genetic biomarkers for SCCs have been identified, although it is not widely accepted that SCCs are of clinical value as a prodromal symptom of PD [49]. 

There is conflicting evidence from individual studies on the etiology of SCCs in PD patients with NCI or CN or the potential correlation between SCCs, clinical characteristics, and neuropsychiatric or affective disorders [26,36,104,107,108]. The interplay between SCCs, MCI, and clinical factors, such as PD duration, severity, or concomitant disease burden, is complex [26,49]. The correlation between depression and SCCs is also not clear. The association between SCCs and global cognition scores in PD-NCI patients can be confounded by depressive symptoms. Patients with SCCs have a higher depression burden compared to patients without SCCs or the deterioration in global cognition over time is associated with higher depression scores in PD patients with SCCs [43,104,105,107,109,110,111]. In some studies, PD patients with SCCs had a higher burden of depression, as well as apathy and anxiety [43,105,107], or the severity of SCCs correlated with the severity of depression, anxiety, and empathy [105]. Therefore, determining pure SCC burden in PD patients may be difficult [36]. Alternatively, the diagnosis of SCCs in PD patients may be indicative of affective disorders and should prompt physicians to carry out further investigation [105]. A limited number of studies have reported lower global cognition scores or greater decline over time in global cognition scores in PD patients with SCCs compared to those without SCCs [43,105,110]. In the cross-sectional study by Pan et al. (2021), the presence of SCCs in newly diagnosed MCI-PD patients was significantly associated with non-motor symptom burden and in the PD-CN group with the time to complete the Stroop color–word test, which is used to assess working memory and attention [26]. It is therefore evident that there are various SCD-related phenotypes. This may imply differences in the underlying pathogenicity of SCCs in PD patients [26,104]. Imaging studies have contributed evidence of differences in PD patients with SCCs versus those without SCCs, such as decreased gray matter density or focal cortical thinning in the frontal, temporal, and occipital areas [112]. 

Other than the potential diagnostic value of the severity of SCCs, the importance of the prompt detection of SCCs in PD patients is evidenced by longitudinal studies on PD patients (up to 7.5 years follow-up) which indicate that SCCs are an independent prognostic factor of an increased risk of conversion to MCI and PDD in PD patients, including PD-CN patients [49,57,105,109,110,111,113,114]. 

### 4.2. Mild Cognitive Impairment

MCI-PD can be defined as the prevalence of NCI without severe effects on daily functioning [40,42] but that affects the activities of daily living and overall quality of life of PD patients [40,44]. The prevalence of MCI-PD ranges from 9–67% [1,40]. The broad range is attributed to the use of different assessment tools and the heterogeneity of PD symptoms as well as the effect of motor fluctuations, neuropsychiatric symptoms, fatigue, sleep disturbances, co-administered treatment (e.g., anticholinergics), and the expected variability of the patient assessments [40]. Visuospatial, EF, and attention deficits and, less often, memory are frequently reported in MCI-PD [1,23,25,35,36,40,42,115,116,117]. Based on increasing evidence over the last decade, MCI-PD is considered an important pre-dementia stage of PD [26]. It is also estimated that 15–40% of PD patients already have MCI at diagnosis, while 20–57% of patients develop MCI up to 5 years after diagnosis [22,35,41,118,119,120,121]. MCI-PD is associated with older age, lower educational level, longer disease duration, higher levodopa equivalent daily dose, more severe motor symptoms, PIGD subtype, poorer quality of life, and higher levels of apathy and depression [122].

MCI can affect one or more neurocognitive functions. Before the establishment of diagnostic criteria for MCI, the results of the ten-year CAMPAIGN study highlighted two phenotypes of NCI in PD patients distinguished into (i) frontal–striatal with dopamine-deficient executive deficits associated with the COMT genotype and (ii) posterior–cortical with not only linguistic and visuospatial deficits but also memory deficits, due to a lack of other neurotransmitters that more closely resemble the pathophysiology of AD and are associated with the APOE E4 genotype, which is consistent with statistically significantly increased odds of developing dementia within 5 years from PD diagnosis [1,40,123,124,125]. The combination of both frontal–striatal and posterior–cortical phenotypes in PD patients is described in the literature [126]. After the establishment of the diagnostic criteria, MCI-PD was classified into (i) subcortical related to psychomotor deficits, reduced attention, and reduced executive and working memory or (ii) cortical related to linguistic deficits, memory deficits, and impaired visuospatial functions [25,40,42]. Similarly to MCI-AD, MCI in PD may affect only memory (amnestic MCI) and/or other neurocognitive functions, i.e., it can be single or multi-domain [1,127,128]. Non-amnestic MCI-PD is characterized by a greater risk of progression to PDD [129]. Comparative studies of PD-CN patients versus amnestic MCI-PD provided evidence of a common pathophysiology that underlies both memory deficits and motor symptoms [129]. Non-amnestic multidomain MCI-PD is characterized by axonal dysfunction, gait disorder, and older age, while female gender and lower overall IQ levels have been associated with amnestic multi-domain MCI-PDI [36,128]. Furthermore, clinical studies demonstrate that non-amnestic multidomain MCI-PD with deficits in verbal fluency, neurocognitive flexibility, and visuospatial functions is associated with faster progression to PDD [41,125,130]. Based on evidence contributed by patients with neurodegenerative diseases, including PD, it is thus proposed that MCI may be refined to one of four subtypes depending on whether memory is impaired and whether single or multiple neurocognitive functions are impaired [131].

Consistent with the PD-related NCI heterogeneity, MCI does not necessarily always deteriorate but may remain stable or return to normal levels within 1 year in approximately 25% of PD patients [36,120]. In the study by Pedersen et al. (2017), the reversal of MCI to normal levels was mainly observed in young patients with short disease duration and mild disease severity [120]. Nevertheless, the long-term risk of PDD remained increased for these patients [36,120]. A relevant meta-analysis including 4011 patients with PD, with a mean age of 58–75 years and mainly of the male gender (61% of patients), reported that among patients with MCI, 20% developed PDD and 28% returned to PD-CN [119]. The use of reliable assessment tools and, possibly, the combination of more than one assessment tool for both global cognition and individual neurocognitive functions, such as neurocognitive batteries, as well as consecutive assessments during patient follow-up, are necessary for the objective assessment of patients who may revert to PD-CN following MCI diagnosis [36,119].

### 4.3. PD Dementia

PD patients have an increased risk in the progression of MCI to dementia compared to non-PD subjects (three- to six-fold greater risk) [132] or subjects with no neurocognitive deficits [41,120]. MCI is associated with a higher risk or shorter time to progress to PDD [23,133,134]. It is estimated that PDD occurs on average within 10 years from PD diagnosis, with approximately 50% of patients developing PPD within this time frame and most PD patients (~80%) developing PDD within 20 years from diagnosis [36,41,55,57,123,134]. In the longest prospective follow-up study of newly diagnosed PD patients lasting 20 years, 74% of patients had died and 83% of survivors had PDD by the end of the study [134]. Other than aging, risk factors for PDD include limited cognitive reserve and gait dysfunction [9,133,134]. Clinical data suggest that the progression of MCI to PDD is a consequence of the interaction of multiple pathologies, which could account for the slower progression of some MCI phenotypes to PDD [36,57,123]. A recent meta-analysis of longitudinal studies in MCI-PD patients who developed PDD highlighted the potential predictive value of impairments in EF and, to a lesser extent, semantic fluency and delayed verbal recall [117]. Because of the association between the latter two neurocognitive functions and the temporal cortex, the progression to PDD in patients with MCI may also be related to a pathophysiology other than Lewy bodies, such as the deposition of beta-amyloid or tau plaques [135,136].

## 5. Kinetic Subtypes of PD and Neurocognitive Impairment

A limited number of comparative studies with small sample sizes suggest that movement tremor is associated with less severe non-motor symptoms [137]. The PIGD subtype is characterized by an overall more progressively deteriorating disease, including more rapid deterioration of motor and neurocognitive status, older age at disease onset, a greater need to initiate levodopa therapy for dementia, or greater risk of dementia onset [12,13,14,15,31,53,54,55,56]. A meta-analysis of prospective cohort studies demonstrated that advanced age, age at PD onset, PIGD, rapid eye movement sleep behavior disorder, hallucinations, orthostatic hypotension, anxiety, APOE ε2, APOE ε4, and EEG theta power > median and alpha power < median were statistically significantly associated with NCI [31]. The PIGD phenotype is not a prognostic indicator for PDD within the subsequent 5 years, though an association between postural/gait disturbances and dementia or MCI has been reported [25,116,130,137]. It can therefore be hypothesized that the coexistence of MCI with mild stiffness may be indicative of a common pathogenetic mechanism [116]. 

Due to the association between the PIGD subtype and NCI, the correlation between individual neurocognitive functions and PD motor symptoms has attracted attention for diagnostic purposes. There is a statistically significant association between IPS and rotation difficulty, namely in the steps required to perform the rotation and rotation duration [138]. Conversely, attention and EF were found to be associated with gait speed, stride length, and rotation range, whereas attention and visuospatial function were related to static balance in pilot studies [139,140]. These results are reinforced by longitudinal studies, which highlight the pace and variability of walking, as well as postural control as predictors of NCI over three years in newly diagnosed PD patients [141]. A recent comparative study of motor symptoms in 193 PD patients with MCI, CN, or PDD reported that gait rate, rotation, and variability were significantly correlated with attention and EF. In contrast, rotation area and balance bounce were related to attention and visuospatial function [139], confirming the existence of common cerebral networks underlying motor and neurocognitive symptoms in PD.

## 6. Social Cognition and PD 

Social cognition (SC) refers to the distinct cognitive and emotional functions that are based on social interactions and not only influence but also determine social behavior [142,143]. It entails the ability to perceive, interpret, and generate responses to the intentions, moods, emotions, and behaviors of other people. SC includes (1) the theory of mind (ToM), which is the ability to understand other people’s intentions (cognitive theory of mind) or their emotional state (affective theory of mind) that collectively explain and predict their behavior [144]; (2) empathy, which is the emotional (affective empathy) or cognitive (cognitive empathy) response to the perceived states of others; (3) social perception, which is the ability to understand social and emotional cues, such as body language and facial emotion recognition, which refers to the ability to recognize and distinguish the emotional state of others based on their facial expressions; and (4) social behavior [145,146]. Facial emotion recognition, as well as SC overall, is intricately related to neural cognition and involves neurocognitive functions, including attention and memory. 

Aging complicates SC functions [34,147,148]. Despite the general belief that complex rather than basic emotions (joy, sadness, anger, surprise, anxiety, and disgust/disgust) are affected by aging [146], a recent meta-analysis of studies in healthy elderly patients demonstrated a reduced and differential ability to recognize both positive and negative basic emotions, with fewer deficits in emotion recognition for happiness and more deficits in fear recognition (in ascending order happiness, disgust, anger, sadness, surprise, and fear) [149]. This finding is important because the recognition of basic emotions is dictated by different neurocognitive functions [150]. Other factors, such as gender and comorbidities, most notably depression, affect SC and the recognition of facial expressions [147,151]. Regardless of comorbidities, NCI can aggravate affective disorders and SC deficits regardless of comorbid conditions [34,99].

While there is still debate on TOM’s association with normal aging since other studies suggest a clear reduction and others provide divergent results, in the case of the relationship between TOM and MCI, things are clearer. L. Clemente et al.’s research showed excellently that there is a complex interaction between cognitive functioning, especially EF, and TOM in healthy elderly people and people with MCI. Specifically, there is a clear reduction in EF and TOM in both states, but it is worse in MCI. The interesting part is that in the case of MCI, there is no significant interaction between EF and TOM, while in normal aging, there is a clear correlation between them. This suggests, on the one hand, that MCI may have a different impact on each parameter but, on the other hand, shows that in the absence of cognitive impairment, different brain domains may interact better with each other [152].

Most of the available studies confirm that PD adversely affects facial emotion recognition, ToM, and empathy [34,147,153,154,155,156]. Compared to healthy controls, PD patients have significant deficits in ToM (both affective and cognitive) and cognitive but not affective empathy, with an emphasis on the recognition of negative versus positive emotions [148,157]. Deficits in the executive function of inhibition are implicated in ToM deficits in PD patients [155]. Clinical parameters implicated in social perceptual difficulties in PD include dopaminergic treatment and the prevalence of motor symptoms, predominantly on the left side [148]. One of the characteristic motor symptoms of PD, freezing, is related to performance in emotional ΤοΜ assessments and anxiety, thus confirming the link between the motor, affective, and neurocognitive symptoms of PD [158,159]. Additionally, transcranial brain stimulation has a beneficial effect on ΤοΜ assessments in MCI-PD patients [33]. 

Similarly, PD patients have reduced, but not absent, or disturbed ability to process facial emotions and experience problems in emotion decoding but not emotion regulation compared to healthy controls [151,154,160]. Since emotion recognition may involve the tendency to reproduce other people’s facial expressions, the hypomimic and reduced movement of facial muscles in PD can further complicate facial emotion assessments [154,161]. MCI-PD patients have a statistically significantly reduced ability to recognize facial emotions compared to PD-CN, who in turn were found to have reduced ability compared to healthy volunteers [162]. Additionally, facial emotion recognition was reduced more progressively in patients with SCD, non-amnestic MCI, and amnestic MCI, compared to health volunteers [162]. The contribution of memory deficits to emotion recognition deficits, both facial and vocal, has been consistently reported in PD patients versus healthy volunteers, regardless of depression and visuospatial deficits [153]. Facial emotion recognition is directly linked to visuospatial functions in PD patients, thus highlighting the importance of working, verbal, and visuospatial memory, attention, and verbal fluency, with varying strengths depending on the emotions assessed [154,162,163]. Despite this potential heterogeneity, negative emotion recognition is consistently affected more than positive emotion recognition [153,154,164]. A subsequent study on emotion recognition in non-depressed MCI-PD, PD-CN, and healthy volunteers demonstrated that PD patients had a reduced ability to recognize facial emotions, especially anger, compared to healthy volunteers, and although healthy individuals and PD-CN focused on the mouth and eyes when visually exploring other people’s faces, MCI-PD patients tended to focus on the center of the face and spent significantly less time scanning the mouth [164]. PN-CN patients also had a reduced ability to focus compared to healthy volunteers. Therefore, inefficient visual exploration may contribute to the impaired ability to recognize facial emotions in PD, while visual scanning of facial emotions is altered even in the absence of NCI.

Alexithymia, defined as the inability to understand and express emotions, is a cognitive–emotional disorder that is increasingly being investigated in patients with neurodegenerative diseases, as the ability to recognize emotions requires that individuals be able to understand their own emotions [165]. Alexithymia is also a risk factor for the manifestation of psychiatric symptoms and correlates with depression, anxiety, and NCI [166,167]. PD patients are twice as likely to develop alexithymia compared to the general population, and alexithymia in PD patients is associated with apathy, depression, anxiety, and impulsivity [168]. Alexithymia is therefore a prevalent non-motor symptom in PD patients, which is reported in up to 30% of PD patients, with a direct impact on social interactions and QoL [169,170,171,172,173]. Various studies have confirmed the association between alexithymia and visuospatial and EF deficits in PD patients compared to healthy volunteers, as well as the correlation between semantic verbal fluency and flow, alexithymia severity, and disease severity [169,170,171,172]. Associations between depression, neurocognitive decline, and alexithymia have also been reported [172,174].

## 7. Conclusions

PD is a complex motor, affective, and cognitive condition. NCI can be prevalent in the prodromal phase of the disease and presents with various phenotypes with detrimental effects on the quality of life of patients and their carers. The establishment of diagnostic criteria for MCI and the availability of tools to assess global cognition and individual neurocognitive functions have contributed significantly to this direction. PD patients usually have multi-domain neurocognitive deficits, as deficits in one neurocognitive function may make patients susceptible to further decline in other functions. 

The up-to-date evidence suggests that NCI in PD is multifactorial, with its onset and progression to PDD reflecting both epidemiological, genetic, and pathobiological factors. NCI is associated with the PIGD subtype more often or more severely. Correlations between motor symptoms and neurocognitive deficits confirm that both motor and non-motor symptoms have common neural substrates and could be exploited prognostically. Due to the correlation between social and neural cognition impairment, the potential effect of alexithymia warrants further investigation. NCI can be confounded by affective disorders in PD patients. This review emphasizes the importance of considering the relationship between motor symptoms, mood disorders, and neuropsychological/social cognition performance in individuals with movement disorders and, in turn, reinforces the need for prompt holistic assessment of PD patients regarding imaging, motor symptom burden, cognition—both neural and social—apathy, anxiety, depression, alexithymia, and genetic background as soon as the diagnosis of PD is made. This is imperative for identifying at-risk PD patients who are more likely to be susceptible to rapid neurocognitive decline and employing strategies to mitigate it. Additionally, it is necessary to standardize the conduct of these assessments at regular intervals during PD patient follow-up.

The heterogeneity among studies on the prevalence, timing of onset, and interactions between affective conditions, neuropsychological functions, and SC performance is partly attributed to the use of different assessment tools. It is therefore important that the tools that are used in PD patients are standardized. The development of tools that encompass both neurocognitive and social cognition assessments is also expected to be valuable. Additionally, MCI diagnostic criteria should be updated to encompass SCCs, which are highly prevalent in newly diagnosed PD patients. SCC onset could represent an at-risk phase, which provides a potentially short window for early and effective cognitive and behavioral interventions. The identification of validated biomarkers that can be used for predicting motor and cognitive outcomes will also contribute both to the prompt diagnosis of PD and the identification of patients who are at higher risk of inferior outcomes or progression to PDD. It is necessary to acquaint clinicians with the fact that PD is a complex multifactorial heterogeneous motor, affective, and cognitive disease. The burden that NCI imposes could be mitigated via the timely identification of PD patients harboring SCCs before their progression to MCI and PDD. The face–name associative memory exam (FNAME), which may be a valuable tool for the prompt diagnosis of AD, may also be used to detect subtle memory deficits in PD patients since memory impairment is the hallmark neurocognitive feature of PD [175,176]. The differentiation of NCI from the confounding effect of apathy, depression, and anxiety is also expected to have significant practical implications in the implementation of effective prompt personalized management strategies, both pharmacological and non-pharmacological, in PD patients as soon as the diagnosis is made.

## Data Availability

Not applicable.

## Abbreviations

PD	Parkinson’s disease
AD	Alzheimer’s disease
TD	Tremor-dominant PD subtype
PIGD	Postural instability and gait disorder PD subtype
ARAD	Akinetic rigid-AD PD subtype
CNS	Central nervous system
NCI	Neurocognitive impairment
EF	Executive functions
MCI	Mild cognitive impairment
PDD	PD dementia
PD CN	PD cognition normal
QoL	Quality of life
SCC	Subjective cognitive complaints
IPS	Information processing speed
CA	Cognitive anosognosia
COMT	Catechol-O-methyltransferase
APOE	Apolipoproteine E
VEGF	Vascular endothelial growth factors
SNCA	a-synouclein
SC	Social cognition
TOM	Theory of mind
